# IL-5 antagonism reverses priming and activation of eosinophils in severe eosinophilic asthma

**DOI:** 10.1016/j.mucimm.2024.03.005

**Published:** 2024-08

**Authors:** Jian Luo, Wentao Chen, Wei Liu, Shan Jiang, Yuan Ye, Rahul Shrimanker, Gareth Hynes, Paul Klenerman, Ian D. Pavord, Luzheng Xue

**Affiliations:** 1 Respiratory Medicine Unit and NIHR Oxford Biomedical Research Centre, University of Oxford, Oxford, United Kingdom; 2 Division of Pulmonary Medicine, Institute of Integrated Traditional Chinese and Western Medicine, West China Hospital, Sichuan University, Chengdu, China; 3 Department of Integrative Medicine, Huashan Hospital, Fudan University, Shanghai, China; 4 Translational Gastroenterology Unit and Peter Medawar Building, University of Oxford, Oxford, United Kingdom

## Abstract

Eosinophils are key effector cells mediating airway inflammation and exacerbation in patients with severe eosinophilic asthma. They are present in increased numbers and activation states in the airway mucosa and lumen. Interleukin-5 (IL-5) is the key eosinophil growth factor that is thought to play a role in eosinophil priming and activation. However, the mechanism of these effects is still not fully understood. The anti-IL-5 antibody mepolizumab reduces eosinophil counts in the airway modestly but has a large beneficial effect on the frequency of exacerbations of severe eosinophilic asthma, suggesting that reduction in eosinophil priming and activation is of central mechanistic importance. In this study, we used the therapeutic effect of mepolizumab and single-cell ribonucleic acid sequencing to investigate the mechanism of eosinophil priming and activation by IL-5. We demonstrated that IL-5 is a dominant driver of eosinophil priming and plays multifaceted roles in eosinophil function. It enhances eosinophil responses to other stimulators of migration, survival, and activation by activating phosphatidylinositol-3-kinases, extracellular signal-regulated kinases, and p38 mitogen-activated protein kinases signaling pathways. It also enhances the pro-fibrotic roles of eosinophils in airway remodeling via transforming growth factor-β pathway. These findings provide a mechanistic understanding of eosinophil priming in severe eosinophilic asthma and the therapeutic effect of anti-IL-5 approaches in the disease.

## INTRODUCTION

Eosinophils are granulocytes that play an important role in chronic inflammatory disorders of the airways such as asthma.^1^ Through release of pro-inflammatory mediators including granule-derived basic proteins, lipid mediators, cytokines, and chemokines, activation of tissue eosinophils contributes to airway inflammation, leading to respiratory dysfunction, airway mucus plugging, and asthma exacerbations.[2], [3] Eosinophilic asthma (EA) is defined by the presence of increased numbers of eosinophils in the blood and airways, and airway eosinophilia is one of the hallmarks of EA.^1^ Blood and sputum eosinophilia is associated particularly with exacerbations in patients with EA.^2^ It has long been noted that the eosinophils from patients with EA are more sensitive in their response to chemokines,^4^ but the understanding of pathogenic alternations of the eosinophils in the disease state remains limited.

Clinical therapies, such as anti-interleukin (anti-IL)-4/13, anti-IL-5, and D prostanoid receptor 2 (DP2) antagonism, that lead to depletion or prevention of migration of eosinophils, can reduce exacerbation frequency and severity in asthma.[5], [6] It has been suggested that type-2 cytokines play critical roles in the pathogenesis of EA, particularly IL-5 which is a key mediator acting at many levels of eosinophil biology. Through binding to IL-5 receptor α-chain (IL-5Rα), IL-5 promotes the proliferation, maturation, migration, survival, and activation of eosinophils.[7], [8], [9] Increased IL-5 levels were detected in EA^10^ and eosinophilia has been observed in IL-5 transgenic mice.^11^ Blockage of IL-5 signaling by anti-IL-5 antibodies or IL-5 depletion reduced lung eosinophilia in animal models,[12], [13] suggesting a crucial role of IL-5 in eosinophilia. Therefore, targeting the IL-5 pathways is an attractive strategy for treating eosinophilic inflammation.^14^ Mepolizumab is a humanized neutralizing antibody against IL-5 approved as adjunct therapy (100 mg subcutaneously every 4 weeks) for patients with severe EA.^5^ It binds to IL-5 and prevents IL-5 from interacting with IL-5Rα on eosinophils, which reduces eosinophil count and risk of asthma exacerbations.^15^ The effects of mepolizumab (100 mg) are associated with only a modest reduction in eosinophil numbers in the airways compared with other biologic therapies and higher dose mepolizumab (750 mg) with similar clinical efficacy[5], [16] suggesting they are primarily mediated via a reduction in eosinophil activation. However, the effect of mepolizumab on the state of activation of mature eosinophils has not been elucidated. It has been suggested that IL-5 is a priming cytokine that enhances the chemotactic response of eosinophils following pre-exposure to low concentrations of IL-5.^8^ In this paper, we use “priming” to describe the effect of IL-5 to promote the potential of eosinophils to respond to further stimuli. A better understanding of this potential IL-5 priming effect on eosinophils could lead to further therapeutic advances for EA.

In this study, we analyzed the activities of eosinophils in severe EA and investigated potential drivers of enhanced eosinophil activation in the disease, particularly the effect of IL-5 by comparison of the cell responses before- and after- mepolizumab treatment. Single-cell RNA sequencing (scRNAseq) was used to explore the mechanisms leading to the change of responses and activations of eosinophils in the disease. Our observations provide new insights into the roles of IL-5 in eosinophilic disorders and biological effect of mepolizumab on eosinophils.

## RESULTS

### Overactivation of eosinophils in EA is suppressed by IL-5 antagonism

**Fig. 1:**
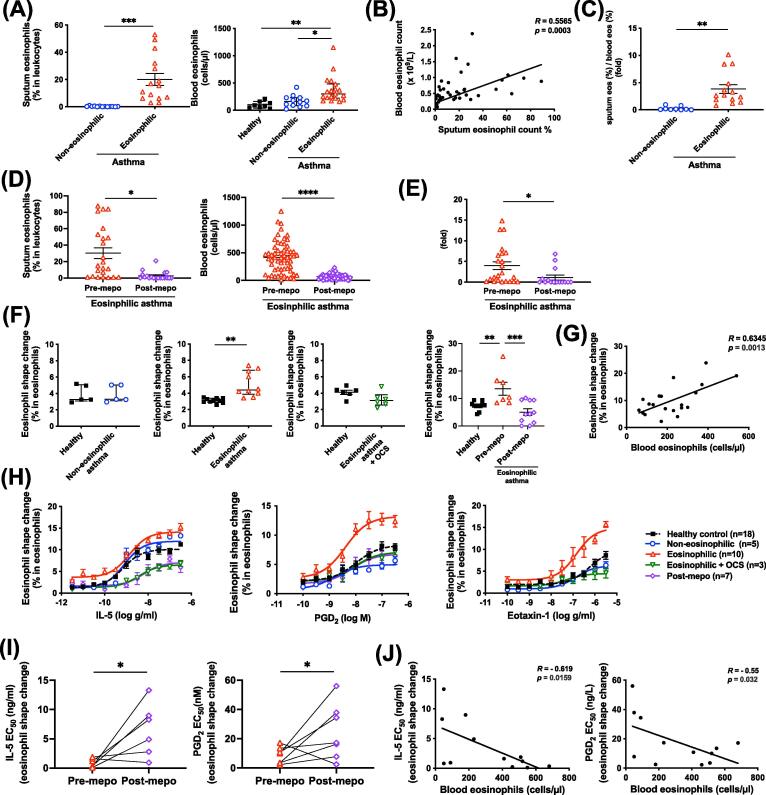
Enhanced eosinophil shape-change in eosinophilic asthma was reversed by mepo. (A–C) Eosinophil levels in sputa and blood samples (A), and their correlation (B) and sputum eosinophils/blood eosinophils ratio (C) in asthma groups. (D, E) Eosinophil levels in sputa and blood samples (D), and their ratio (E) before and after mepo treatment for 4 weeks. (F) Baseline eosinophil shape-change in blood from different asthma phenotypes and medical treatments paired with healthy controls. (G) Correlation of baseline eosinophil shape-change and blood eosinophil counts. (H) Blood eosinophil shape-change in response to various concentrations of IL-5, PGD_2,_ or eotaxin-1 from different asthma conditions and medical treatments. (I, J) Potencies of IL-5 and PGD_2_ in the induction of blood eosinophil shape-change before and after mepo treatment in eosinophilic asthma (I) and their correlation with blood eosinophil counts (J). *R* indicates Spearman’s correlation coefficient. *p < 0.05, **p < 0.01, ***p < 0.001 and ****p < 0.0001. EC50 = half-maximal effective concentrations; IL = interleukin; mepo = mepolizumab; OCS = oral corticosteroids; PGD_2_ = prostaglandin D_2_.

As the name suggests, EA is characterized by abnormally high eosinophil counts both in the patient’s blood and airways (Fig. 1A and Supplementary Table 1), which show positive correlation (Fig. 1B). Furthermore, EA also showed increased recruitment of eosinophils from the peripheral blood to the airways as the ratio of sputum/blood eosinophil frequency was significantly increased in EA (3.83 ± 0.8, *n* = 14) compared with that in non-EA (0.253 ± 0.11, *n* = 9, *p* = 0.002) (Fig. 1C). Clinical treatment with mepolizumab reduced sputum and blood eosinophil counts in the patients with EA (Fig. 1D), together with the ratio of sputum/blood eosinophil frequency (Fig. 1E). To investigate the mechanism of airway eosinophilia in asthma patients, we cross-sectionally compared the activities of eosinophils between asthma phenotypes. Mepolizumab clinical treatment was used as a tool to dissect the role of IL-5 involvement.

We first tested eosinophil shape-change (Supplementary Fig. 1A), a biomarker of eosinophil migration,^17^ in whole blood under physiological conditions using flow cytometry (Supplementary Fig. 1B). Increased ratio of eosinophil shape-change was detected in the blood from patients with EA compared with healthy or non-EA controls (Fig. 1F). Treatment of patients with EA with oral corticosteroids (OCS) prednisolone or mepolizumab reduced the baseline levels of eosinophil shape-change (Fig. 1F). The levels of eosinophil shape-change (% in eosinophils) were positively correlated with the blood eosinophil counts (Fig. 1G). Treatment of whole blood with established eosinophil chemoattractant, such as IL-5, prostaglandin D_2_ (PGD_2_) and eotaxin-1, further enhanced eosinophil shape-change in a dose-dependent manner (Fig. 1H and Supplementary Fig. 2A). The eosinophil shape-change in response to IL-5 positively correlated with the sputum eosinophil counts although this did not achieve statistical significance (Supplementary Fig. 2B). The specificity of these chemokines could be confirmed by inhibition of IL-5 with an anti-IL-5 antibody, inhibition of DP2 with a DP2 antagonist (CAY10471), or inhibition of CCR3 (a receptor for eotaxins) with a CCR3 antagonist (SB328437) (Supplementary Fig. 2C). The level of eosinophil shape-change in response to chemokines in blood from EA were significantly higher than those from non-EA and healthy individuals (Fig. 1H and Supplementary Fig. 2A). Such enhanced responses in the patients with severe EA were completely lost, even dropping to levels lower than the healthy controls, after treatment of the patients with OCS or mepolizumab, which was also observed by Stein et al.^18^ in their previous study. However, the corticosteroid, dexamethasone, did not inhibit the level of eosinophil shape-change induced by chemoattractants *ex vivo* (Supplementary Fig. 2C), suggesting that corticosteroids do not directly inhibit chemoattractant-induced cell shape-change but target upstream gene/protein regulation. The half-maximal effective concentrations of IL-5 or PGD_2_ to induce eosinophil shape-change were increased after mepolizumab treatment (Fig. 1I), which were negatively correlated with the blood eosinophil counts (Fig. 1J). In contrast to eosinophils, neutrophil shape-change in response to the chemokine IL-8 was not changed in EA compared with that in healthy subjects (Supplementary Fig. 2D).

**Fig. 2:**
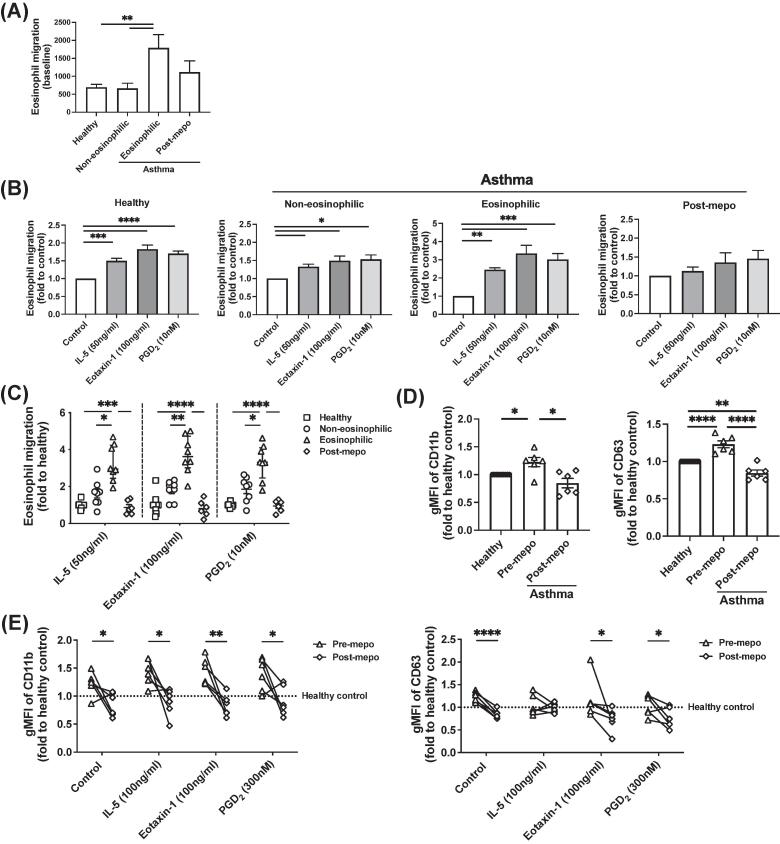
Enhanced eosinophil migration and activation in eosinophilic asthma were reversed by mepo. (A–C) Cell migration in eosinophils isolated from blood from different asthma conditions and mepo treatment without chemoattractant (A) or induced by IL-5, eotaxin-1, and PGD_2_ (B) measured with chemotaxis, and comparison of responses between patient groups (C). Fold in (C) indicates the fold difference from the mean value of healthy samples. (D, E) Expression of CD11b and CD63 in blood eosinophils in asthma groups before and after mepo treatment without (D) or with stimulation with IL-5, eotaxin1, or PGD_2_ for 1 h (E) detected with flow cytometry. *p < 0.05, **p < 0.01, ***p < 0.001 and ****p < 0.0001; *n* = 6-16 for (A and B). CD = cluster of differentiation; gMFI = geometric mean fluorescence intensity; IL = interleukin; mepo = mepolizumab; PGD_2_ = prostaglandin D_2_.

To confirm the observation in the shape-change assays, we next examined cell migration using the eosinophils isolated from the different asthma groups (Figs. 2A–C and Supplementary Fig. 3A). The eosinophils from EA showed higher migration capacity under baseline conditions (Fig. 2A). IL-5, eotaxin-1, and PGD_2_ showed chemoattractive effects on eosinophils in chemotaxis assays, with significantly higher sensitivity seen with eosinophils from patients with EA than healthy or non-EA (Figs. 2B and 2C). Moreover, mepolizumab treatment significantly reduced the effects of these chemokines on eosinophils in patients with EA.

We further examined eosinophil activation with the activation marker cluster of differentiation (CD)11b^19^ and degranulation marker CD63^20^ using flow cytometry (Figs. 2D and 2E). The levels of CD11b and CD63 on the surface of eosinophils were higher in the patients with severe EA compared with healthy controls, which were also reduced after mepolizumab treatment (Fig. 2D). Increased CD11b expression was observed in eosinophils after stimulation with IL-5, eotaxin-1 or PGD_2_, but the increase of CD63 expression was less significant (Fig. 2E and Supplementary Fig. 3B) although the releases of granular proteins, eosinophil-derived neurotoxin and eosinophil cationic protein, were detected in IL-5 or PGD_2_ treated eosinophils (Supplementary Fig. 3C). Mepolizumab treatment abrogated the increase of CD63 expression completely.

### Enhanced eosinophil activation in EA is not associated with chemokine receptor expression levels

**Fig. 3:**
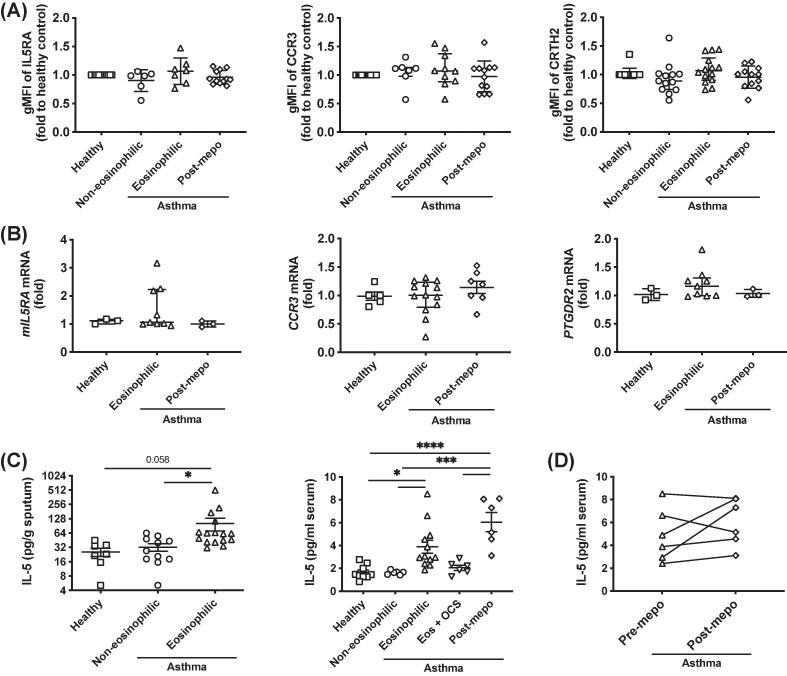
Expression of chemokine receptors in eosinophils and interleukin-5 levels in patients was not altered by mepolizumab. (A, B) Comparison of the expression of chemokine receptors in eosinophils from different patient groups at protein level detected with flow cytometry in whole blood (A) or at transcriptional level (*mIL5RA* for membrane-bound IL-5RA, *CCR3* for CCR3, and *PTGDR2* for CRTH2) measured with qPCR in purified eosinophils (B). (C) Levels of IL-5 in sputum or serum from different asthma conditions and medical treatments determined with Luminex. (D) Effect of mepolizumab on serum IL-5 levels in paired samples. *p < 0.05, ***p < 0.001 and ****p < 0.0001. eos = eosinophilic; gMFI = geometric mean fluorescence intensity; IL = interleukin; IL-5RA = IL-5 receptor α-chain; mepo = mepolizumab; mRNA = messenger ribonucleic acid; OCS = oral corticosteroids; qPCR = quantitative polymerase chain reaction.

To understand whether the enhanced eosinophil activation in EA was due to the upregulation of the chemokine receptors in these eosinophils, we compared the expression levels of IL-5 receptor (including subunits IL5RA and CSF2RB), CCR3 and DP2 in eosinophils from different asthma conditions at protein level using flow cytometry (Fig. 3A and Supplementary Fig. 4A). The levels of these receptors were very similar between healthy individuals and patients with non-eosinophilic or EA. Mepolizumab treatment in EA patients did not alter the expression of these receptors. Quantitative polymerase chain reaction (qPCR) studies also confirmed that these receptors (including soluble IL-5 receptor *sIL5RA*) were not changed at transcriptional level in EA patients or after mepolizumab treatment (Fig. 3B and Supplementary Figs. 4).

We further compared the concentrations of the chemokine ligands between the patient groups. Small increases of IL-5 in sputum and significant increases in serum were detected in EA compared with healthy or non-eosinophilic individuals (Fig. 3C). No significant difference was detected in eotaxin concentrations in EA, although eotaxin-2 was increased in serum from non-eosinophilic samples (Supplementary Fig. 5A). The concentration of PGD_2_ in serum was undetectable (data not shown), but was increased in sputum in asthma, regardless of phenotype.^21^ We also checked other eosinophil activation-related cytokines, particularly β common (βc) family proteins, including granulocyte-macrophage colony-stimulating factor (GM-CSF), IL-3, and thymic stromal lymphopoietin （TSLP） in blood serum (Supplementary Fig. 5A). No difference in these cytokines was detected in either eosinophilic or non-eosinophilic patients. The increase of IL-5 in the sera was inhibited by OCS but not by mepolizumab treatment (Figs. 3C and 3D). Similarly, mepolizumab did not demonstrate any inhibitory effect on the serum levels of other cytokines (Supplementary Fig. 5B). Therefore, IL-5 is likely to be an important factor for enhanced eosinophil activation in EA.

### Single-cell transcript profiles of blood eosinophils from severe EA before and after IL-5 inhibition

**Fig. 4:**
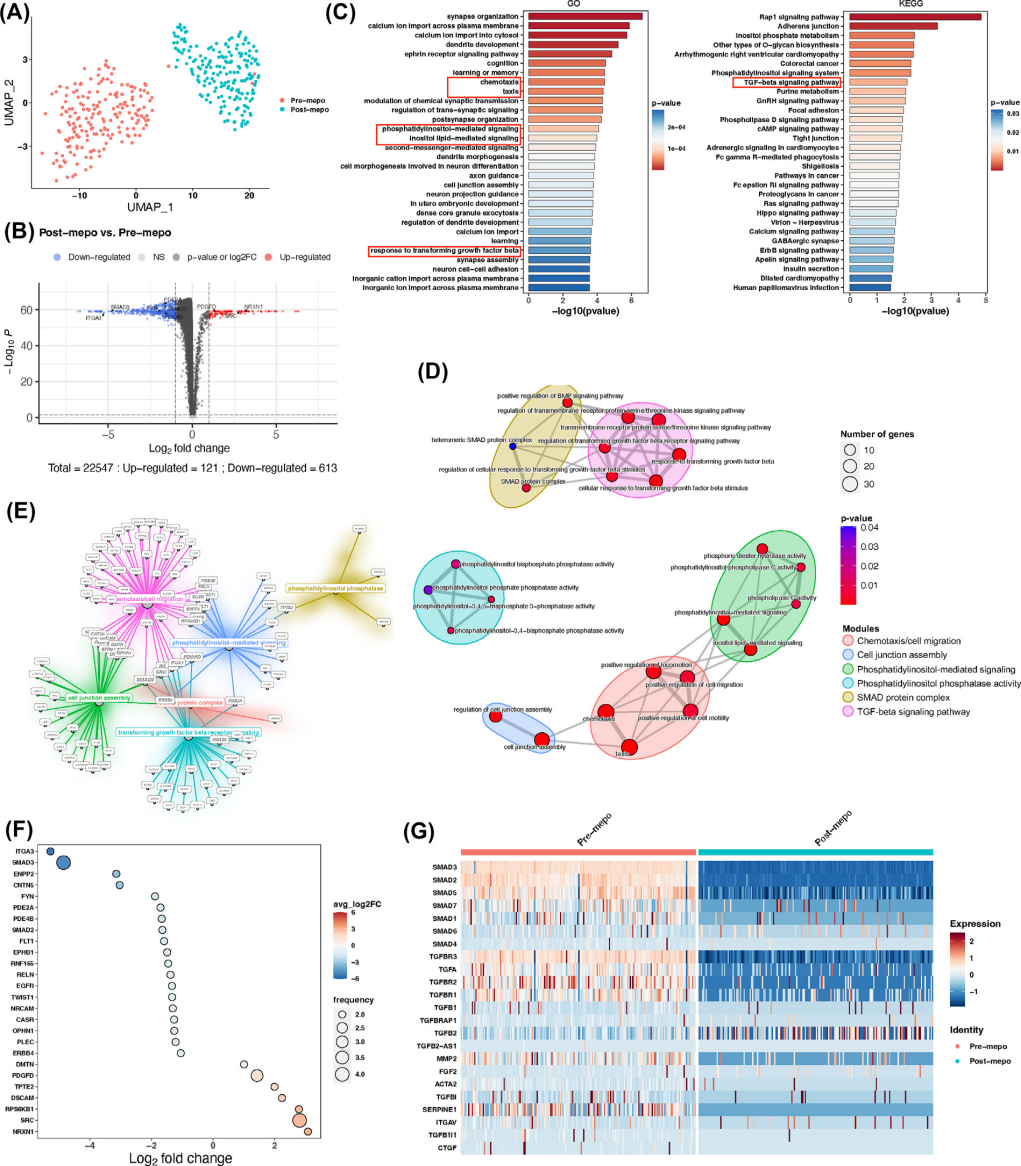
Effect of mepolizumab on the transcriptional profiles of eosinophils from patients with severe eosinophilic asthma detected by single-cell ribonucleic acid sequencing. (A) UMAP visualized the transcriptional distribution of pre- and post-mepo eosinophils. (B) Differential gene expression analysis in pre- and post-mepo eosinophils where genes were highlighted in blue (downregulated), gray (no change), black (no significant change), or red (upregulated). (C) Pathway enrichment analysis showing top list of pathways involved in the genes that are detected in the RNAseq. (D) Pathways with semantic similarity clustered into six functional modules. (E) Correlation of genes in the six functional modules. (F) Connecting node genes regulated by mepolizumab among the six functional modules. (G) Heatmap showing the comparison of expression levels of key genes involved in TGF-β pathway between pre- and post-mepo eosinophils. FC = fold change; GO = Gene ontology; KEGG = Kyoto Encyclopedia of genes and Genomes; mepo = mepolizumab; RNAseq, ribonucleic acid sequencing; scRNAseq = single-cell ribonucleic acid sequencing; UMAP = Uniform Manifold Approximation and Projection; TGF-β = transforming growth factor-β.

To broadly investigate the potential molecular mechanism underlying the enhanced activations of eosinophils mediated by IL-5 in EA, we conducted eosinophil transcriptional analysis. Paired eosinophil samples from EA patients before (pre-mepo) and after (post-mepo) mepolizumab treatment were sorted for scRNAseq (Fig. 4 and Supplementary Fig. 6). The sorted eosinophils expressed eosinophil rather than neutrophilic specific markers genes, especially high in *CCR3*, *IL5RA,* and *EPX* (Supplementary Fig. 6A). Dimensional reduction analysis showed that eosinophils from pre-mepo and post-mepo were clustered distinctively on Uniform Manifold Approximation and Projection (Fig. 4A) and Principle Component Analysis (Supplementary Fig. 6B and 6C). Compared with pre-mepo, eosinophils from post-mepo were associated with 121 upregulated genes and 613 downregulated genes, among which *SMAD3, SMAD2* and *ITGA3* were highly downregulated (Fig. 4B). Pathway enrichment analysis revealed 710 gene ontology (GO) categories and 41 Kyoto Encyclopedia of Genes and Genomes (KEGG) pathways, among which chemotaxis, phosphatidylinositol-mediated signaling, and response to transforming growth factor-β (TGF-β) signaling pathway appeared in the top list (Fig. 4C and Supplementary Fig. 6D). GO semantic similarity analysis identified relevant categories/pathways associated with the above-mentioned top cellular functions and enrichment map clustered them into six functional modules: (1) chemotaxis/cell migration, (2) cell junction assembly, (3) phosphatidylinositol-mediated signaling, (4) phosphatidylinositol phosphatase activity, (5) SMAD protein complex, (6) TGF-β signaling pathway (Fig. 4D). The differentially expressed genes (DEGs) in these modules were partially connected between each other (Fig. 4E). By focusing on those connecting node genes, we found two interesting genes (*SMAD2* and *SMAD3*) that were downregulated in post-mepo eosinophils (Fig. 4F). Since SMAD2 and SMAD3 are key intracellular effectors of TGF-β signaling, pathway-associated genes including the SMAD family, TGF-β receptor, TGF-β, and TGF-β regulated genes were compared before and after patient treatment with mepolizumab. Among these, *SMAD2*, *SMAD3*, *SMAD5*, *TGFBR2*, *TGFBR3* and *MMP2* were significantly downregulated by mepolizumab (Fig. 4G and Supplementary Fig. 6E–G).

### Activation of signal pathways in eosinophils from EA is inhibited by IL-5 antagonism

**Fig. 5:**
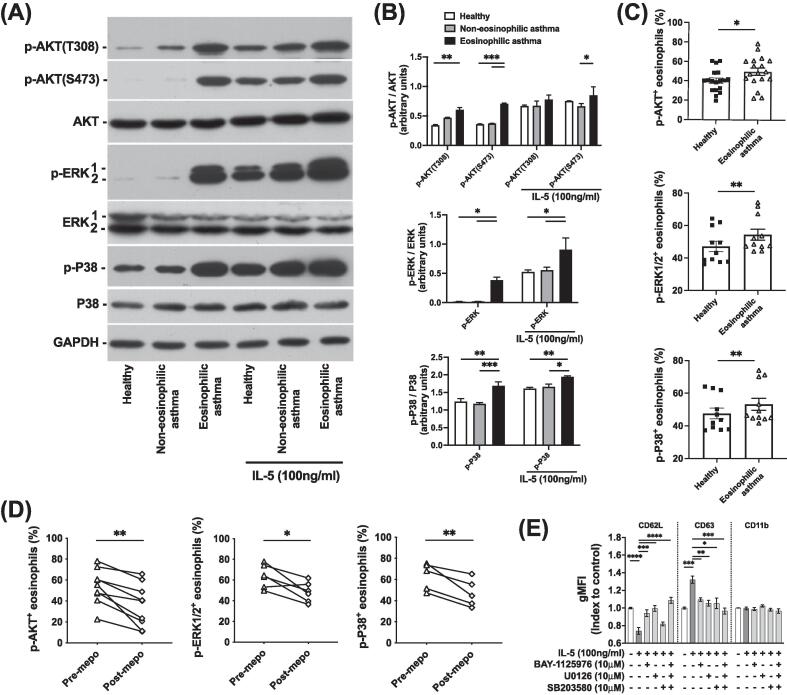
Phosphorylation on phosphatidylinositol-3-kinases, mitogen-activated protein kinase, and p38 signal pathways in eosinophils from eosinophilic asthma was inhibited by mepolizumab. (A, B) Phosphorylation on Akt, ERK, and p38 in eosinophils from different asthma groups before and after IL-5 stimulation was detected with Western blot (A), and quantification of band intensities in the Western blot (B). (C, D) Frequencies of p-Akt, p-ERK, and p-p38 positive eosinophils in total eosinophils compared between healthy and eosinophilic asthma patients (C), and between before and after mepolizumab treatment in eosinophilic asthma patients (D) measured with Phosflow in fresh blood. (E) Effects of signal pathway inhibitors on the activation of eosinophils in response to IL-5 stimulation determined by the levels of CD62L, CD63, and CD11b under flow cytometry. *p < 0.05, **p < 0.01, ***p < 0.001 and ****p < 0.0001. *n* = 3 for (B), *n* = 7 for (E). AKT = protein kinase B; CD = cluster of differentiation; ERK = extracellular signal-regulated kinases; gMFI = geometric mean fluorescence intensity; IL = interleukin; MAPK = mitogen-activated protein kinase; mepo = mepolizumab; p- = phosphorylated-; p38 = p38 mitogen-activated protein kinases; PI3K = phosphatidylinositol-3-kinases.

Phosphatidylinositol-3-kinases (PI3K), extracellular signal-regulated kinases (ERK), and p38 mitogen-activated protein kinases (p38) have been reported to be involved in human eosinophil activation, migration, and survival.[22], [23] To further confirm the signaling activities detected in pre/post-mepolizumab eosinophils by scRNAseq, we examined the phosphorylation of relevant kinases in the eosinophils from EA. The phosphorylation of protein kinase B, also known as AKT (p-AKT at both threonine 308 and serine 473 for PI3K pathway), ERK1/2 (p-ERK1/2 for ERK pathway) and p38 (p-p38 for p38 pathway) measured with Western blot was increased in the eosinophils isolated from EA compared with those from healthy or non-eosinophilic controls (Fig. 5A). The intensity level of p-AKT bands detected in EA was ∼2-fold of those in healthy control and non-EA, while p-ERK1/2 was ∼20 fold and p-p38 was ∼1.5-fold (Fig. 5B). IL-5 stimulation enhanced the phosphorylation of these kinases in eosinophils across all donors (Figs. 5A and 5B). Increases of p-AKT, p-ERK1/2 and p-p38 in the eosinophils from EA were also confirmed using phosphor flow cytometry (Fig. 5C and Supplementary Fig. 7A). Treatment with mepolizumab reduced the frequencies of p-AKT^+^, p-ERK1/2^+^ or p-p38^+^ eosinophils in the blood in the patients with severe EA (Fig. 5D).

To dissect the roles of these signal pathways in eosinophil activation by IL-5 in EA, BAY1125976 (AKT inhibitor), U0126 (ERK inhibitor), and SB203580 (p38 inhibitor) were employed to probe the relative contributions of different pathways to eosinophil activation. Stimulation with both IL-5 or phorbol 12-myristate 13-acetate (PMA)/ionomycin caused activation marker CD62L shedding and degranulation marker CD63 increase on the eosinophil surface (Fig. 5E and Supplementary Fig. 7B). BAY1125976, U0126, and SB203580 partially reversed, and combination of the three inhibitors almost completely blocked the response, indicating that these three pathways contribute to the eosinophil activation and degranulation induced by IL-5 or PMA/ionomycin, although the effects on CD11b, another activation marker, were undetectable.

### Enhanced response to TGF-β in eosinophils from EA contributes to airway remodeling

**Fig. 6:**
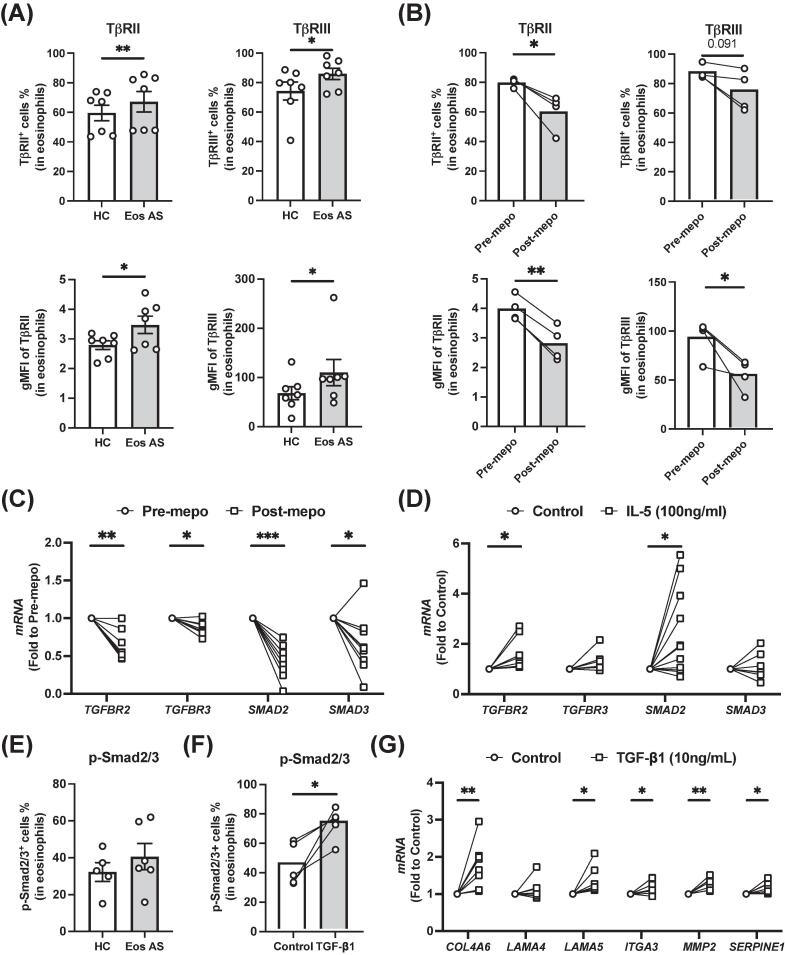
Transforming growth factor-β pathway in eosinophils from patients with eosinophilic asthma and its potential role in tissue remodeling. (A, B) Expression of TβRII and TβRIII in eosinophils from HC, Eos AS (A), before (pre-mepo), or after (post-mepo) mepo treatment (B) measured with flow cytometry. (C, D) mRNA levels of *TGFBR2*, *TGFBR3*, *SMAD2* and *SMAD3* in the eosinophils from eosinophilic asthma before or after mepolizumab treatment (C) or from healthy donors before or after IL-5 treatment (D) measured by qPCR. (E, F) Levels of phosphorylation of smad2/3 detected by Phosflow assays in eosinophils from HC or Eos AS (E), or before or after TGF-β1 treatment (F). (G) Regulation of the expression of extracellular matrix proteins by TGF-β1 in isolated eosinophils determined by qPCR. *p < 0.05, **p < 0.01, ***p < 0.001, ****p < 0.0001. Eos AS = eosinophilic asthma; gMFI = geometric mean fluorescence intensity; HC = healthy control; IL = interleukin; mepo = mepolizumab; mRNA = messenger ribonucleic acid; p- = phosphorylated-; qPCR = quantitative polymerase chain reaction; TGF-β = transforming growth factor-β.

To confirm IL-5-dependent activation of the TGF-β signaling pathway in eosinophils from EA patients indicated by the scRNAseq, we measured the expression of TGF-β receptors, TβRII and TβRIII, in eosinophils from various patient groups *ex vivo* (Figs. 6A and 6B and Supplementary Fig. 8A). Both TβRII^+^ and TβRIII^+^ eosinophils were increased in the blood from severe EA compared with healthy controls (Fig. 6A). This was reversed by mepolizumab treatment (Fig. 6B). The expression levels of TβRII and TβRIII were also upregulated in EA and downregulated by mepolizumab (Supplementary Fig. 8A), suggesting that IL-5 contributes to the upregulation of TGF-β receptors. This regulation was supported by the evidence from qPCR (Fig. 6C), although no significant upregulation of *TGFBR3* messenger ribonucleic acid (mRNA) by IL-5 was observed *in vitro* (Fig. 6D). We also found that expression of *TGFB2* mRNA in eosinophils was reduced by mepolizumab and increased by IL-5 stimulation (Supplementary Fig. 8B).

We next addressed the intracellular phosphorylation-dependent proteins of TGF-β signaling pathway. The binding of TGF-β to its receptor leads to the phosphorylation of SMAD proteins (SMAD2 and SMAD3) and the subsequent formation of SMAD transcription factor complex. Consistent with the results of scRNAseq, downregulation of *SMAD2* and *SMAD3* mRNA by mepolizumab was also confirmed in qPCR (Fig. 6C). Furthermore, IL-5 stimulation upregulated the expression of *SMAD2* (but not *SMAD3*) in eosinophils (Fig. 6D). However, no significant increase of phosphorylated SMAD2/3 protein in the eosinophils from EA was detected *ex vivo* (Fig. 6E and Supplementary Fig. 8C).

To further investigate the role of TGF-β signaling in eosinophils, we stimulated isolated eosinophils with TGF-β1. TGF-β1 significantly increased the phosphorylation of SMAD2/3 (Fig. 6F and Supplementary Fig. 8C) and downregulated CD62L but did not alter the level of CD63 (Supplementary Fig. 8D). Furthermore, TGF-β1 stimulated eosinophils to produce extracellular matrix (ECM) including *COL4A6* and *LAMA5*, *ITGA3*, and ECM proteolysis associated proteins including *MMP2* and *SERPINE1* (Fig. 6G), thus suggesting a potential contribution of the activated eosinophils in airway tissue remodeling in EA.

## DISCUSSION

In EA, eosinophils are not only increased in numbers in both blood and airways reflecting enhanced recruitment and migration, but also altered in terms of their behavior. In this study, we demonstrated that the eosinophils from EA patients possessed greater capacity for migration and activation than those from healthy individuals and non-eosinophilic patients. They were more sensitive to different chemoattractant stimuli with enhanced activation in disease-relevant signal pathways. This altered eosinophil behavior was neither associated with the expression levels of chemoattractant receptors nor the blood concentrations of these chemoattractants except for IL-5. The key contribution of IL-5 to the enhanced eosinophil activation in EA is supported by the effect of mepolizumab as a clinical treatment, which not only reduced eosinophil counts in blood and airways but also reduced pro-inflammatory capacity of eosinophils in eosinophilic patients. Our results demonstrated, for the first time, the effect of mepolizumab on eosinophil priming in the disease. The transcriptional changes following mepolizumab treatment in eosinophils further confirmed that IL-5 mediated eosinophil priming and activation, as well as potentially contributing to tissue remodeling and airway fibrosis. These observations provide a better insight into the mechanisms underlying EA and the beneficial effects of mepolizumab therapy.

Previous studies have suggested that several growth factors or chemokines (eotaxins, IL-2/3/5/8, GM-CSF, and PGD_2_) could prime eosinophils,[8], [24], [25], [26], [27] but it is still unclear which factor dominates the priming effect of eosinophils in asthma and if priming is reversible. Our data indicated that IL-5 plays a dominant role in the eosinophil priming in EA since, among these factors, only the increase of IL-5 was detectable in our cohort of EA patients while IL-5 antagonism blocked the priming completely. IL-5 has been identified as an eosinophil-specific cytokine in the 1980s,^7^ and can be produced by type-2 immune cells including type-2 T helper 2, type-2 cytotoxic T cell 2, and group 2 innate lymphoid cells, mast cells, natural killer T cells, and eosinophils.[28], [29], [30], [31] It plays a vital role in the terminal differentiation, growth, survival, and activation of eosinophils, and possesses eosinophil chemotactic activity.^32^ IL-5^-/-^ and IL-5Ra^-/-^ mice exhibit impaired eosinophilopoiesis and enhanced survival of intracranial worms, indicating that IL-5 is critical for eosinophil function as effector cells.^33^ It has been well-documented that IL-5 is a key driver of EA^34^ and many other disorders of eosinophilia.[10], [35] We confirmed the elevation of IL-5 in EA in our cohort and detected a much higher level of IL-5 in sputum compared with that in blood. Mepolizumab reduced eosinophil counts, and reversed eosinophil priming and activities. It has also been reported that pre-treatment with mepolizumab could reduce eosinophil increase as well as eosinophil degranulation triggered by allergen challenge, although could not reduce the upregulation of some activation markers by allergen.^36^ In our study, the concentration of IL-5 was not changed by mepolizumab in the patients, indicating that its inhibitory effect on eosinophils is not via reduction of IL-5.

IL5Rα and a common β chain (βc).^37^ IL-5Ra specifically binds to IL-5 and induces the recruitment of βc to IL-5R.^38^ The βc is a signal-transducing molecule shared with IL-3R and GM-CSFR.^39^ The expression of IL-5 receptor and other chemokine receptors in eosinophils was not significantly altered in EA^40^ or by mepolizumab treatment. Even in the previously published study, increase of IL5Rα in eosinophils was observed after long (12 weeks) mepolizumab treatment.^18^ Therefore, modulated levels of these receptors are unlikely to be the reason for eosinophil priming.

Transcriptomic analysis of eosinophils from mepolizumab-treated patients provided us with a profile of gene expression regulated by IL-5 antagonism under physiological conditions, which helped to better understand the effects of IL-5 on eosinophil priming in the disease. To our knowledge, this represents the first single-cell transcriptomic dataset for human *ex vivo* blood eosinophils from patients with asthma undergoing mepolizumab treatment. Previous bulk RNAseq on purified human eosinophils only addressed *in vitro* stimulation through common β-chain receptor.^41^ Recently a scRNAseq on human tissue-resident T helper 2/eosinophils investigated the pro-inflammatory roles of these cells in eosinophilic esophagitis.^42^ Another scRNAseq analysis of mouse eosinophils from different organs suggested distinctive subpopulations.^43^ The analysis of our single-cell transcriptomic dataset revealed a broad range of transcriptional changes induced by mepolizumab, including gene sets associated with cell migration, activation, mitogen-activated protein kinase (MAPK) signaling, and TGF-β/SMAD-mediated tissue remodeling.

IL-5 stimulation induces rapid tyrosine phosphorylation of various cellular proteins including the IL-5R βc, SH2/SH3-containing proteins, Btk-associated molecules, Jak/signal transducer and activator of transcription (STAT), PI3K, MAPK, and activates their downstream signaling pathways.[44], [45] It has been reported that PI3K/AKT, MAPK/ERK, and p38 MAPK were involved in IL-5-induced eosinophil shape-change.[17], [23] Our data also detected pronounced changes of PI3K, MAPK/ERK, and p38 signaling pathways associated with IL-5/mepolizumab in eosinophils through comparison of pathway activations between either healthy and EA or pre- and post-mepolizumab under physiological condition, which provided for the first time direct evidence of these pathways involved in IL-5 priming under asthma condition. PI3K and MAPK are important signaling pathways that control cell survival, proliferation, differentiation, metabolism, and motility. P38 signaling may contribute to the chemotaxis of eosinophils and neutrophils.^22^ In our patient cohort, IL-5 antagonism not only reduced eosinophil counts, activities, and their response to different chemokines but also decreased the activation of PI3K, ERK, and p38 signaling pathways. Furthermore, blockage of PI3K, ERK, and p38 pathways almost completely prevented eosinophil activation in response to IL-5. Therefore, we can conclude that the priming effect of IL-5 in EA is mediated through these three pathways. The tumor necrosis factor (TNF)/NF-κB pathway has been reported to mediate the activation of esophageal eosinophils in eosinophilic esophagitis.^42^ It would be interesting to investigate whether TNF/NF-κB pathway also contributes to eosinophil priming in our future studies.

Airway remodeling in severe EA is a complex process involving goblet cell metaplasia, subepithelial matrix protein deposition, airway smooth muscle hypertrophy, and angiogenesis.^46^ Many cytokines (e.g. IL-4, IL-13), growth factors (e.g. TGF-β, vascular endothelial growth factor), and proteases (e.g. matrix metalloproteases, plasminogen activator inhibitor-1) have been implicated in causing airway remodeling,^47^ among which TGF-β is one of the best-characterized mediators. TGF-β can be produced by many cell types in the lungs, including epithelial cells, fibroblasts, smooth muscle cells and eosinophils.^48^ Binding of TGF-β to TGFβRII triggers the recruitment of TGFβRI and phosphorylation of SMAD2 and SMAD3. The phosphorylated SMAD2 and SMAD3 form the transcription factor complex with the co-mediator SMAD4, which ultimately translocates to the nucleus to regulate the expression of pro-fibrotic genes including collagens, integrins, connective tissue growth factor, plasminogen activator inhibitor-1, and matrix metalloproteases.^49^ In patients with severe asthma, airway remodeling results in increased airway wall thickness and enhanced deposition of ECM proteins in the bronchial subepithelial basement membrane.^50^ Increased levels of TGF-β in serum and bronchoalveolar lavage^51^ and SMAD2/3/4 in bronchial mucosa^52^ have been observed in asthma. Notably, the level of airway remodeling is positively correlated with bronchial mucosal eosinophil numbers, and bronchial eosinophils are believed to be the main source of TGF-β in asthma.^50^ However, the link between IL-5 and the pro-fibrotic roles of eosinophils is still unclear. Our data show that IL-5 enhanced eosinophil response to TGF-β by priming the cells to express more TGF-β receptors and SMAD proteins in EA, which were downregulated by mepolizumab treatment. TGF-β promoted eosinophils to express ECM proteins, suggesting that IL-5 could induce the pro-fibrotic roles of eosinophils through TGF-β/SMAD pathway. A comparison of bronchial biopsies from patients with asthma found that the thickness of tenascin and airway smooth muscle mass were significantly reduced after treatment with biologics targeting IL-5 or IL-5 receptor,[50], [53] associated with the reduction of TGF-β^+^ eosinophils, which is consistent with our finding that TGF-β production in eosinophils was elevated by IL-5 while decreased by mepolizumab. Therefore, our results demonstrate that eosinophil priming by IL-5 in EA not only enhances the migration, survival, and activation of eosinophils but also promotes the contribution of eosinophils to airway remodeling.

In summary, our study highlights the multifaceted role of IL-5 in the activation of eosinophils in EA. Using mepolizumab, we demonstrated that IL-5 is a dominant driver of eosinophil priming in the disease. IL-5 enhances eosinophil migration, survival, activation, and also the eosinophil responses to other stimulators through activation of PI3K, ERK, and p38 signal pathways. It also enhances the pro-fibrotic roles of eosinophils in airway remodeling via TGF-β pathway. Overall, these findings provide an important mechanistic insight into eosinophil priming in eosinophilic disorders and the therapeutic effect of anti-IL-5 treatment.

## METHODS

### Patients and clinical samples

Patients with asthma or healthy subjects were recruited from Oxford University Hospitals, Oxford, United Kingdom (Supplementary Table 1). Severe asthma with a sputum eosinophil count of >3% (eosinophilic) or <3% (non-eosinophilic) was recruited based on the American Thoracic Society/European Respiratory Society definition.^54^ Severe eosinophilic patients treated with mepolizumab but without OCS history were selected for paired sample collection (pre-mepo: before mepolizumab injection *vs.* post-mepo: 1 month after 1^st^ 100 mg mepolizumab (GlaxoSmithKline) injection). Severe eosinophilic patients treated with OCS (>7 days) were also enrolled as clinical treatment controls. The protocol was approved by South Central-Oxford B Research Ethics Committee (18/SC/0361). Written informed consent was obtained from each donor before sample collection.

Blood samples were collected in ethylenediaminetetraacetic acid tubes and used directly for flow cytometry analysis, for granulocyte preparation for scRNAseq, or used for eosinophil isolation for chemotaxis assays, Western blot, Enzyme-linked immunosorbent assay or qPCR.

Sputum was induced and processed according to standardized methodology published by European Respiratory Society Task Force.[55], [56] Briefly, sputum was induced with nebulized saline solution (3%–5%) after pre-treatment with salbutamol (GlaxoSmithKline). Selected sputum plugs were dispersed with 4× the sputum weight of phosphate buffer saline (SIGMA), and part of the sample was used to prepare supernatant for Luminex assays. The remaining sample was further dispersed by adding the same volume of 0.2% dithiothreitol (SIGMA), and then filtered for cells for slide eosinophil counting.

### Eosinophil isolation

Whole blood was layered on top of Lymphoprep™ (StemCell Technologies) for density gradient separation. The lower red blood cell layer was collected and mixed with an equal volume of 3% dextran (SIGMA) solution. After precipitation at room temperature in the dark for 30 min, the granulocyte-rich supernatant was harvested and centrifuged. Remaining red blood cells in the cell pellet were lysed using sterile ice-cold H_2_O. Eosinophils were further purified using MACS® CD16 microbeads (Miltenyi Biotec) according to the manufacturer’s instructions. The purity of CD16-negative eosinophils was confirmed with a BD LSRII flow cytometer (BD Biosciences).

### Flow cytometry

For blood eosinophil counting, whole blood was labeled with antibody panel 1 (Supplementary Table 2) for 30 minutes in dark at 4°C. After red blood cell lysis and washing, samples were analyzed using a flow cytometer (BD LSRII). Eosinophils were identified as side scatter^high^CD193^+^CD16^-^ cells.

For cell surface biomarkers and receptor determination, whole blood with or without stimulation with IL-5 (100 ng/mL, Miltenyi Biotec), eotaxin-1 (100 ng/mL, R&D Systems), PGD_2_ (300 nM, Enzo Life Science) or TGF-β1 (10 ng/mL, SIGMA) for 40 minutes was stained with antibody panel 2 (for biomarkers, Supplementary Table 2) or panel 3 (for receptors, Supplementary Table 2) for 30 minutes at 4°C, followed by red blood cell lysis and fixation with a BD FACS Lysing Solution (BD Biosciences), and then analyzed using a BD LSRII flow cytometer. In some experiments, whole blood was pre-treated with AKT1/2 inhibitor (BAY1125976, 10 µM, Cayman), MEK1/2 inhibitor (U0126, 10 µM, Calbiochem) and p38 inhibitor (SB203580, 10 µM, Calbiochem) for 1 hour.

When geometric mean fluorescence intensity (gMFI) was retrieved, a paired non-staining sample was used to measure the background gMFI of the target receptor/protein each time, and the fold change of gMFI in the stained samples to the background gMFI was calculated and presented.

### Eosinophil shape-change assay

Whole blood with or without incubation with various concentrations of IL-5, PGD_2_, or eotaxin-1 in the presence or absence of anti-IL-5 antibody (3 µg/mL, R&D systems), CAY10471 (1 µM, Abcam), SB328437 (3 µM, Tocris) or dexamethasone (100 nM, Tocris) at 37°C 5% carbon dioxide for 1 hour (IL-5 and PGD_2_) or 10 minutes (eotaxin-1) were fixed with BD Cytofix™ (BD Biosciences). Red blood cells were lysed by an RBC lysis buffer (Qiagen), followed by analysis using a flow cytometer. Eosinophils were gated by their intrinsic autofluorescence in the phycoerythrin (PE) channel. The shape-change was defined by the increased shift in forward scatter compared with a control.

### Chemotaxis assay

Isolated eosinophils were resuspended in Roswell Park Memorial Institute 1640 media (SIGMA) to a final concentration of 4 × 10^5^ cells/mL. The chemoattractant medium (29 μL) and eosinophil suspension (25 μL) were loaded onto the lower and upper chambers of a 5-μm pore-size 96-well ChemoTx plate® (Neuro Probe) respectively. After 1 hour incubation at 37°C (5% carbon dioxide), the cell suspension in the lower chambers was harvested and mixed with same volume of Cell Titer-Glo® Luminescent Cell Viability Assay kit (Promega). Luminescence was quantified with an EnVision Multilable Reader (PerkinElmer).

### Phospho flow cytometry (Phosflow)

Phosphoproteins (p-AKT, p-ERK1/2, p-p38 and p-Smad2/3) in eosinophils were measured using Phosflow (BD Biosciences) following BD Phosflow^TM^ protocol for human peripheral blood mononuclear cells. Cell pellets from fixed whole blood samples (stimulated with or without 10 ng/mL TGF-β1 for 30 minutes) were permeabilized with Perm Buffer II or III based on the specific antibodies (BD Biosciences) and then stained with anti-p-AKT, p-ERK1/2, p-p38 or p-Smad2/3 antibodies conjugated with Alexa Flour 647 or PE (BD Biosciences). After washing, the cells were analyzed in a BD LSRII flow cytometer. Eosinophils were gated by side scatter^high^ and their autofluorescence in PE or fluorescein isothiocyanate (FITC) channel.

### Luminex assays

The concentrations of cytokines and chemokines in serum or sputum were measured using a Luminex Performance Assay kit (Bio-techne) following the manufacturer’s instructions. Results were obtained with a Bio-Plex 200 System (Bio-Rad).

### qPCR

Total RNA of the isolated eosinophils was extracted using a Rneasy Mini Kit (Qiagen). cDNA was prepared using a High-Capacity cDNA Reverse Transcription Kit (Applied Biosystems). qPCR was conducted using Fast SYBR Green Master Mix (ThermoFisher) in a CFX96 Real-Time PCR Detection System (Bio-Rad Laboratories). *EEF1A1* was used as a reference gene. Primers used are listed in Supplementary Table 3.

### scRNAseq

scRNAseq was performed following Smart-seq 2 protocol^57^ with slight modification for eosinophils. Granulocyte-rich supernatants were prepared as aforementioned in eosinophil isolation from the blood of the patients with severe EA with three paired samples before and after mepolizumab treatment, and then were stained with antibody against CD16 (eBioscience) together with Annexin-V (BioLegend) and 4',6-diamidino-2-phenylindole (DAPI, Sigma). Single eosinophils were sorted from the stained samples in 2 μL of Lysis Buffer in 96-well plates by using BD FACSAria (BD Biosceinces, 96 cells/donor at each visit). After cDNA library construction with Rnasin® Plus Ribonuclease Inhibitor (Promega), pooled samples were sent to Novogene (United Kingdom) Company for sequencing using a NovaSeq platform (Illumina).

Quality control was performed to exclude doublet (number of unique genes > 18,000) and dead (percentage of reads that mapped to the mitochondrial genome > 1%) cells, which resulted in 100% of cells (96 cells per sample) for the subsequent gene expression analysis. One patient dataset was removed due to a change in the patient’s medical condition (severe influenza and respiratory symptoms at the post-mepo timepoint). The data was then analyzed using Seurat package (version 4.1.1) in R (version 4.2.0) for dimensional reduction on Principle Component Analysis and clustering on Uniform Manifold Approximation and Projection. DEGs between clusters were identified (adjusted *p* < 0.05 and absolute Log2 fold change > 1) and presented in volcano plot using EnhancedVolcano package (version 1.14.0). Pathway enrichment analysis was conducted using clusterProfiler package (version 4.4.4), and GO semantic similarity analysis was performed to cluster the functionally similar pathways. DEGs in the identified pathway were presented in network using igraph package (version 1.3.2), and node genes were shown in bubble plot using ggplot2 package (version 3.4.0). Genes associated with the identified pathways were visualized by heatmap, violin plot, and feature plot using Seurat package (version 4.1.1). The raw genomic data is available at the Oxford Research Archive.

### Western blot

Isolated eosinophils were resuspended in a serum-free Roswell Park Memorial Institute medium for ∼1 hour. After stimulation with IL-5 (100 ng/mL) for 1 hour, the cells were lysed in 200 μl ice-cold lysis buffer (50 mM Tris-hydrochloric acid pH 8.0, 150 mM sodium chloride, 1 mM ethylenediaminetetraacetic acid, 1% SDS, 1% Triton X-100, and protease-inhibitor mixture, Merck), and then sonicated for 3 seconds. Nuclei and debris were removed by centrifugation. The protein concentrations were measured with Pierce BCA Protein Assay Kit (Thermo Fisher). The samples were fractionated by sodium dodecyl-sulfate polyacrylamide gel electrophoresis and then electrophoretically transferred to a polyvinylidene difluoride (PVDF) membrane (Merck), and probed with antibodies (Cell Signaling Technology) as indicated in the results. The intensity of immune-positive bands was quantified using Quantity One software (Bio-Rad).

### Statistical analysis

Date was analyzed by using 1-way analysis of variance, followed by Tukey’s test or *t* test. Correlation R values were calculated by using the nonparametric Spearman correlation test. Half-maximal effective concentrations were determined by using Nonlinear regression of XY analyses in Prism v10.1.1 (LLC). Values of *p* ≤ 0.05 were considered statistically significant. Data are presented as means ± standards of the mean.

## AUTHOR CONTRIBUTIONS

JL, WC and LX designed the experiments; JL, WC and WL performed the experiments and acquired, analyzed, and interpreted the data; SJ and YY were involved in part of the experiments; RS, GH and IDP provided patients’ phenotype identification, medical record analysis, and sample collection; PK, IDP and LX acquired funding for the project, and conceived and supervised the project; JL and LX wrote and edited the manuscript.

## DECLARATIONS OF COMPETING INTEREST

The authors have no competing interests to declare.

## FUNDING

This Research was funded by grants from the National Institute for Health Research Oxford Biomedical Research Centre (to JL, WC, PK, IDP and LX) and the Wellcome Trust (WT222426/Z/21/Z to PK). For the purpose of Open Access, PK has applied a CC BY public copyright licence to any Author Accepted Manuscript version arising from this submission.
