# Supplementary material for: IL-5 antagonism reverses priming and activation of eosinophils in severe eosinophilic asthma
Source: Mucosal Immunol. 2024 Aug;17(4):524–36. doi: 10.1016/j.mucimm.2024.03.005 (PMC11649845; doi:10.1016/j.mucimm.2024.03.005)
Supplement: Supplementary data 1 [file mmc1.pdf]

SUPPLEMENTARY INFORMATION

IL-5 antagonism reverses priming and activation of eosinophils in severe eosinophilic asthma

Luo J, Chen W, Liu W, Jiang S, Ye Y, Shrimanker R, Hynes G, Klenerman P, Pavord ID and Xue L.

SUPPLEMENTARY METHODS

Eosinophil cytospin and Rapi-Diff staining

Isolated eosinophils (50-100 × 10^3) cells were loaded into a cytofunnel and spun to form a monolayer of cells on a slide. After fixation with methanol, the cells were stained with Rapid Diff II staining kit (Atom Scientific) and mounted with DPX mountant (Sigma Aldrich) according to the manufacturer's instructions. Images were obtained under a Zeiss Axioskop 2 microscope.

Enzyme-linked immunosorbent assay (ELISA)

The concentrations of eosinophil-derived neurotoxin (EDN) and eosinophil cationic protein (ECP) in the supernatants from isolated eosinophils after treatment with medium, IL-5 (1 μg/ml) or PGD_2 (1 μM) were measured using ELISA kits (Hycult Biotech for EDN and Abcam for ECP) following the manufacturer's instructions. The results were detected by an EnVision Multilabel Plate Reader (PerkinElmer).

SUPPLEMENTARY TABLES

Supplementary Table 1. Study subjects (Mean ± SD)

Characteristic	Healthy	Severe asthma		*p*-value
		Non-eosinophilic	Eosinophilic	
	n=29	n=18	n=57	
Age (y)	31.73 ± 10.12	55.59 ± 18.08	52.57 ± 18.13	< 0.001
Gender (M/F)	10/19	9/9	29/28	0.332
Atopy (%)	13.8	55.6	59.6	< 0.001
BMI	23.34 ± 2.77	28.69 ± 4.04	29.46 ± 7.98	< 0.001
FEV1 (% pred)	102.50 ± 14.96	83.73 ± 42.25	76.37 ± 31.80	0.001
FeNO (ppb)	24 ± 23	48 ± 39	54 ± 68	0.062
Sputum eosinophils (%)	0.25 ± 0.22	1.1 ± 0.7	23.55 ± 17.1	< 0.001
Blood eosinophils (10^9/l)	0.16 ± 0.10	0.24 ± 0.15	0.49 ± 0.26	< 0.001
ICS dose (BDP)	0	1145 ± 684	1581 ± 3365	0.660

BMI, body mass index; FeNO, fractional exhaled nitric oxide; FEV1, forced expiratory volume in one second; SD, standard deviation.

Supplementary Table 2. Antibodies used for flow cytometry

Antigen	Clone	Supplier	Panel number
CD11b	CBRM1/5	BioLegend	2
CD16	CB16	eBioscience	1,3
CD16	3G8	BioLegend	2
CD62L	DREG-56	BioLegend	2
CD63	H5C6	BioLegend	2
CD125 (IL5RA)	REA705	Miltenyi Biotec	3
CD193 (CCR3)	5E8-G9-B4	eBioscience	1,3
CRTH2	BM16	Miltenyi Biotec	1,3
TβRII	W17055E	BioLegend	3
TβRIII	polyclonal	Proteintech	3

Panel 1 for blood eosinophil counting;
Panel 2 for eosinophil activation biomarkers;
Panel 3 for receptors.

Supplementary Table 3. Primers used for q-PCR

Gene	Primer
CCR3	5’-AGCACACAAGCCAGGTTCC-3’<br>5’-GCAGGCCCACGTCATCATAG-3’
COL4A6	5’-GGCTCTACTGGTTTATCGGGA-3’<br>5’-CCTTGAGTTCCATTACAGCCATC-3’
EEF1A1	5’-TCCTGTGAAACCCAGTGTCTT-3’<br>5’-TTCATTTATTGTAGTGAGCAAGTTTGT-3’
GAPDH	5’-AGCCACATCGCTCAGACAC-3’<br>5’-GCCCAATACGACCAAATCC-3’
ITGA3	5’-GCGCAAGGAGTGGGACTTAT-3’<br>5’-TGAAGCTGCCTACCTGCATC-3’
LAMA4	5’-GTTGCGCTCCTGGCTACTAT-3’<br>5’-TATGGTTGGGCAGTCCATGC-3’
LAMA5	5’-CCCACCGAGGACCTTTACTG-3’<br>5’-GGTGTGCCTTGTTGCTGTT-3’
mIL5RA	5’-GCAGCAGTGAGCTCCATGTG-3’<br>5’-AGGGCTTGTGTTCATCATTTCCC-3’
MMP2	5’-GCTACGATGGAGGCGCTAAT-3’<br>5’-GGGCAGCCATAGAAGGTGTT-3’
PTGDR2	5’-CCTGTGCTCCCTCTGTGC-3’<br>5’-TCTGGAGACGGCTCATCTG-3’
SERPINE1	5’-CGCAAGGCACCTCTGAGAA-3’<br>5’-TCACCAAAGACAAGGGCCAG-3’
sIL5RA	5’-GCAGCAGTGAGCTCCATGTG-3’<br>5’-TTCAGATACGGTGTGGGGCAG-3’
SMAD2	5’-ACCAAGCACTTGCTCTGAAAT-3’<br>5’-ACGACCATCAAGAGACCTGG-3’
SMAD3	5’-TGAGGCTGTCTACCAGTTGAC-3’<br>5’-TGGTCACAGTCTGTCTCCTG-3’
TGFB2	5’-CTTTGGATGCGGCCTATTGC-3’<br>5’-CTGCTGTGCTGAGTGTCTGA-3’
TGFBR2	5’-CTGTCTGTGGATGACCTGGC-3’<br>5’-CTCCCACTGCATTACAGCGA-3’
TGFBR3	5’-CCTATCCCGCAAGCTGACAT-3’<br>5’-CCCAGATTATCGAGGCGTCC-3’

SUPPLEMENTARY FIGURES

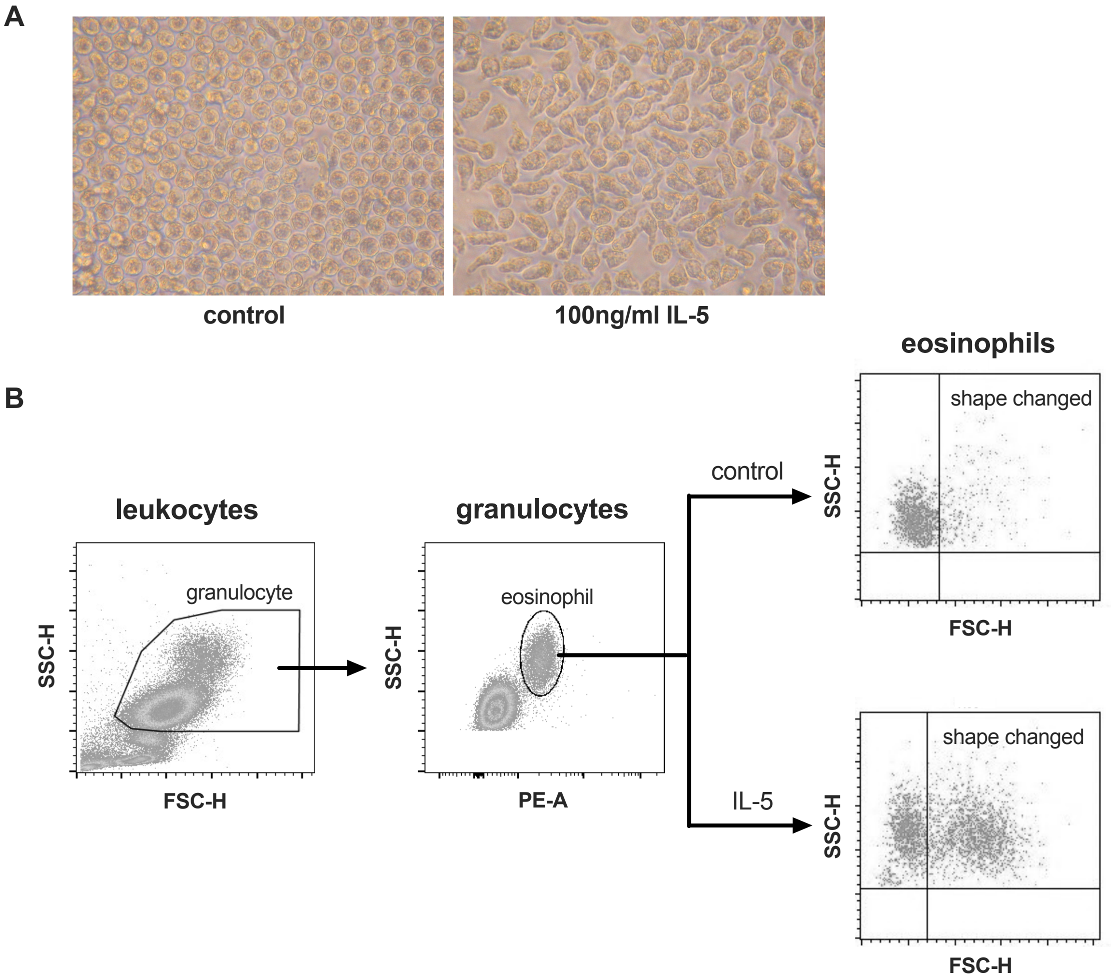

Supplementary Fig. 1 Eosinophil shape-change. **(A)** Representative image of eosinophil shape-change induced by IL-5 in purified eosinophils. **(B)** Gating strategy of eosinophil shape-change in whole blood. Granulocytes were gated as side scatter high (SSC^{high}) cells, and then eosinophils were gated from granulocytes by their autofluorescence (PE^{high}). Eosinophil shape changes were determined by the shift in forward scatter compared to control.

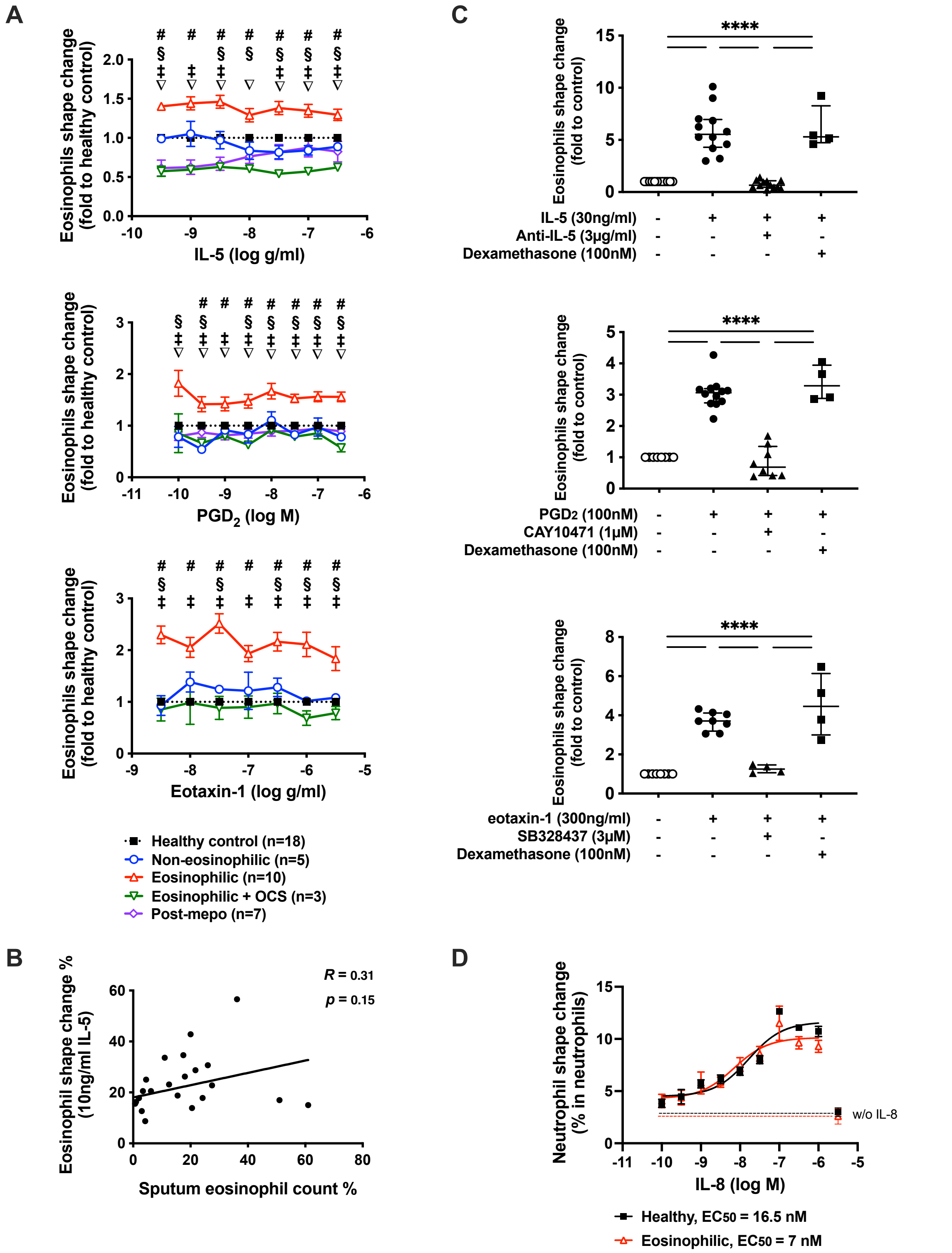

Supplementary Fig. 2 Eosinophil shape-change in response to chemoattractants were inhibited by blockage of these chemoattractants. **(A)** Comparison of eosinophil shape-change in response to IL-5, PGD_2 or eotaxin-1 from different asthma groups and medical treatments to that from healthy controls (statistical analysis of data in Figure 1h). **(B)** Correlation of eosinophil shape-change in response to IL-5 and sputum eosinophil counts. **(C)** Eosinophil shape-change in response to IL-5, PGD_2 or eotaxin-1 was inhibited by anti-IL-5 antibody, CAY10471 or SB328437 respectively but not by dexamethasone. **(D)** Neutrophil shape-change in response to IL-8 was not enhanced in eosinophilic asthma. *R* = Spearman's correlation coefficient. ****p<0.0001; p<0.05 for # Eos vs OCS, § Eos vs Non-eos, ‡ Eos vs HC and ∇ Eos vs Post-mepo in **(A)**. n=6 for **(D)**.

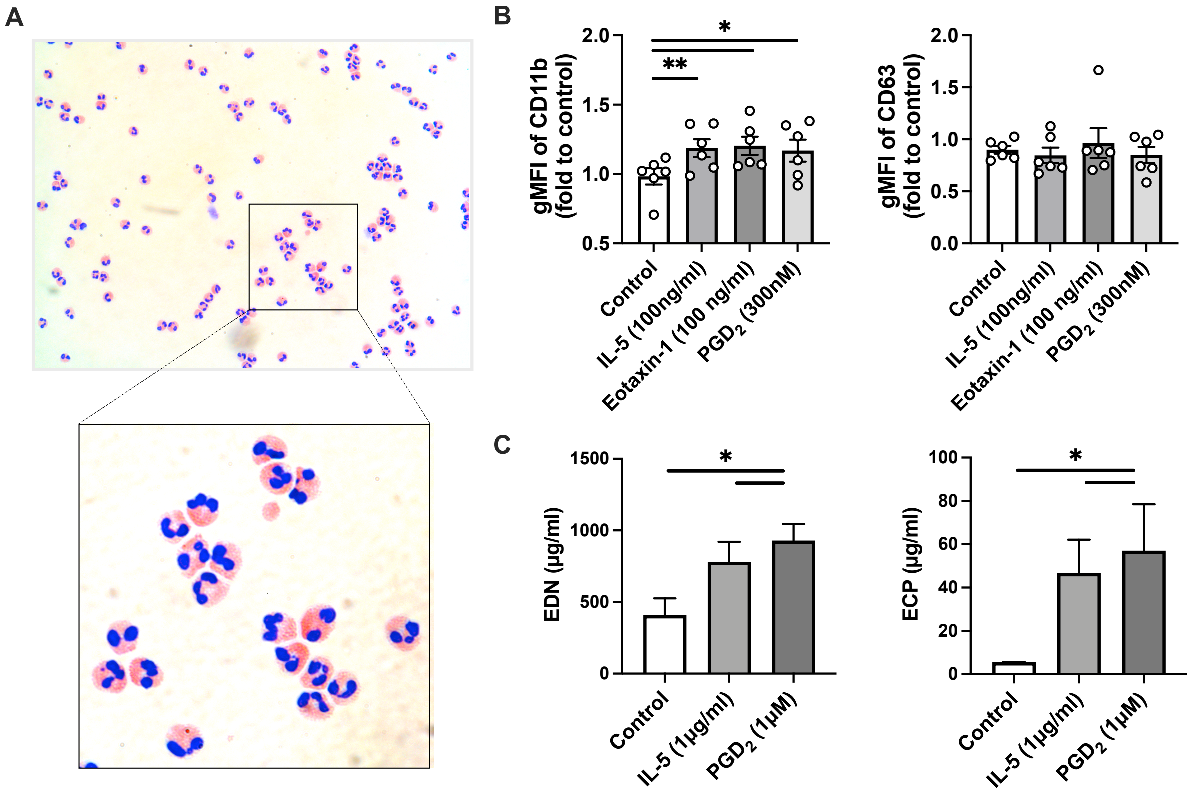

Supplementary Fig. 3 Eosinophils were activated by IL-5, eotaxin and PGD_2. **(A)** Isolated eosinophils were stained with Rapi-Diff II. **(B)** Expression of CD11b and CD63 in blood eosinophils from severe eosinophilic asthma (before mepolizumab treatment) with or without stimulation with IL-5, eotaxin-1 or PGD_2 for 1 h. **(C)** Concentration of eosinophil-derived neurotoxin (EDN) and eosinophil cationic protein (ECP) released by isolated eosinophils treated without or with IL-5 or PGD_2 measured with ELISA. *$p<0.05$, **$p<0.01$. n=10 for **(C)**.

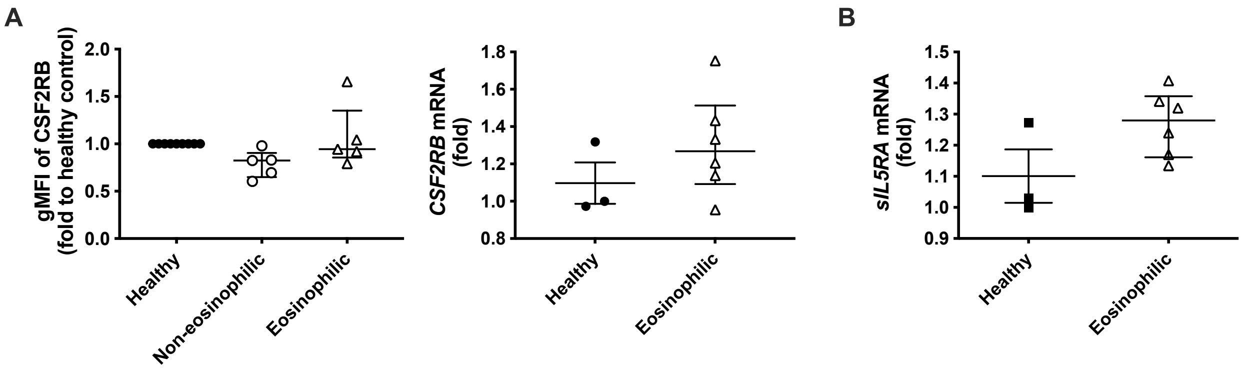

Supplementary Fig. 4 Expression of IL-5 receptor subunit CSF2RB **(A)** and soluble IL5RA **(B)** in healthy control, non-eosinophilic and eosinophilic asthma detected using flow cytometry and qPCR.

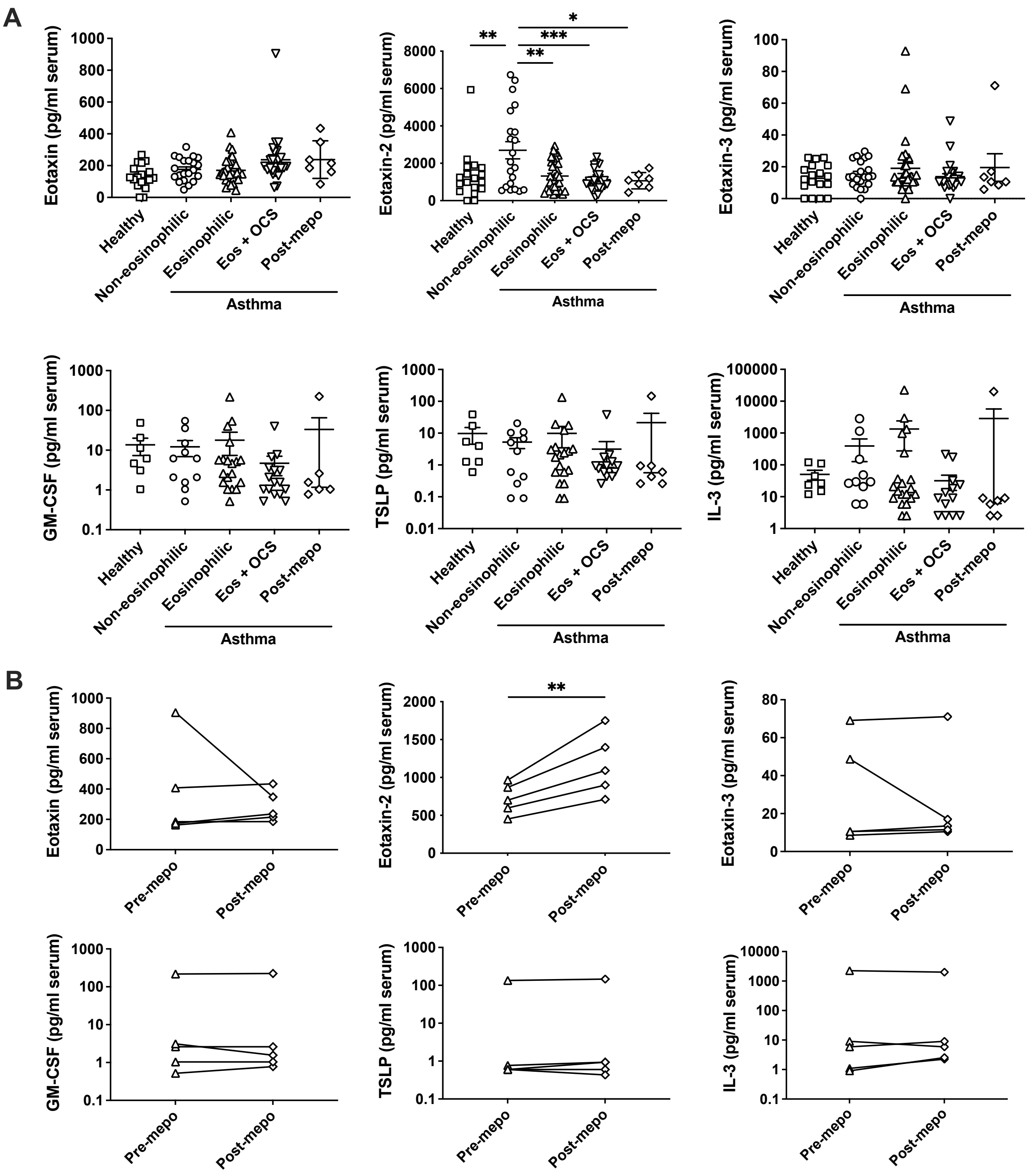

Supplementary Fig. 5 Blood serum concentrations of selected eosinophil chemoattractants and cytokines were not changed in asthma phenotypes or by medical treatments. **(A)** Comparison of the concentrations of eotaxin 1/2/3, GM-CSF, TSLP and IL-3 in the sera from different asthma phenotypes and after OCS or mepolizumab treatments measured with Luminex. **(B)** Comparison of the concentrations of eotaxin 1/2/3, GM-CSF, TSLP and IL-3 in paired serum samples from patients with eosinophilic asthma between before and after mepolizumab treatment. **p*<0.05, ***p*<0.01, ****p*<0.001.

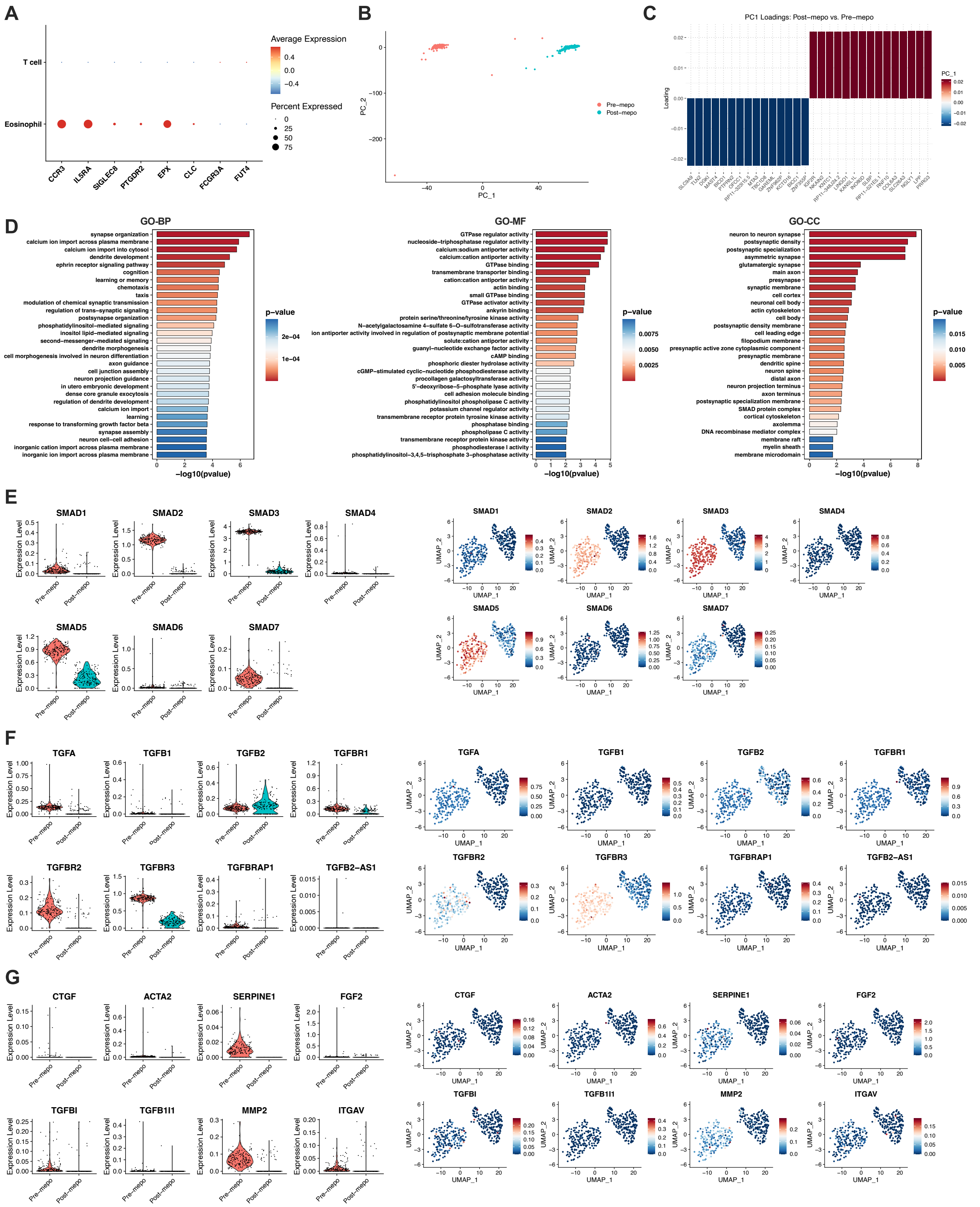

Supplementary Fig. 6 Effect of mepolizumab on the transcriptional profiles of eosinophils from the patients with severe eosinophilic asthma detected by scRNAseq. **(A)** Dot plot of eosinophil-specific or neutrophil-specific genes expressed by sequenced eosinophils. Expression levels by T cells (data from 10X datasets) were used as controls for comparison. **(B)** PCA visualised the transcriptional distribution of Pre- and Post-mepo eosinophils. **(C)** Top 30 genes contributing to PC1**. (D)** Pathway enrichment analysis showing the top 30 pathways in GO-BP, GO-MF, and GO-CC. **(E-G)** Violin plots or UMAP visualised the regulation of genes in SMAD family **(E)**, TGF-β and receptors **(F)** and downstream of TGF-β **(G)** by mepolizumab.

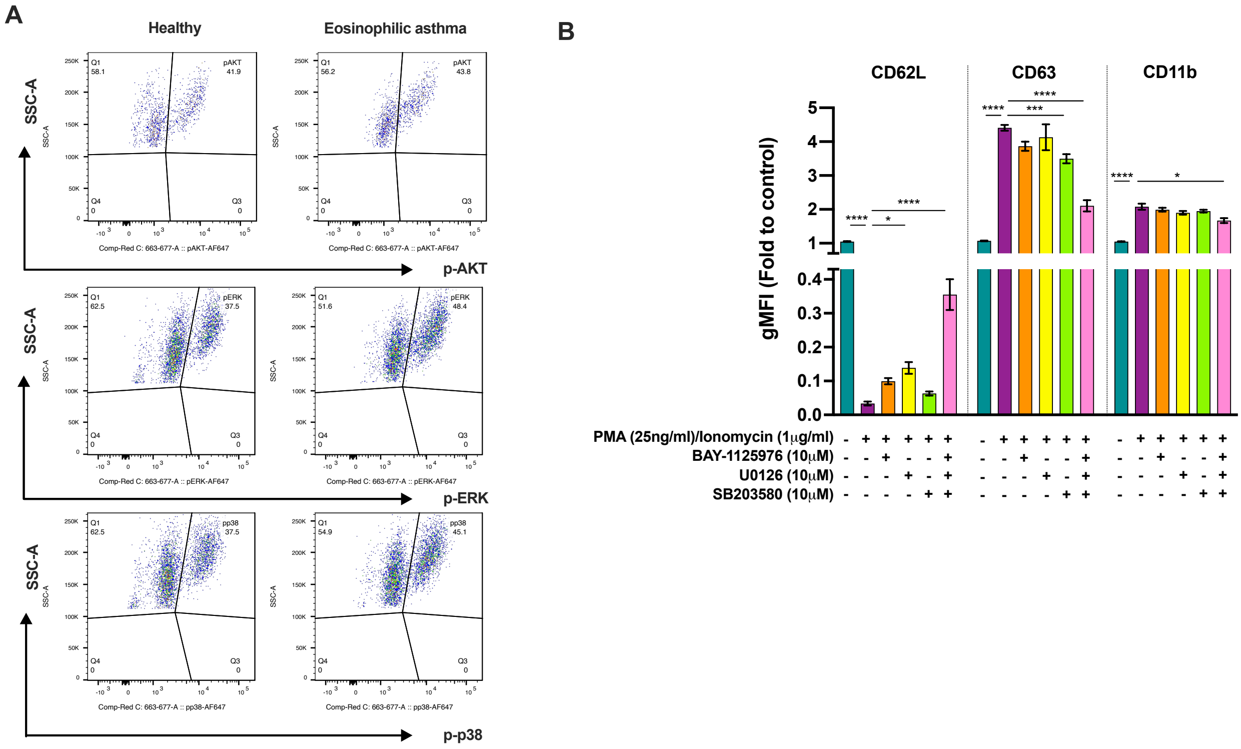

Supplementary Fig. 7 Signal pathways contribute to the activation of eosinophils. **(A)** Comparison of the ratios of p-AKT, p-ERK and p-p38 positive eosinophils between healthy and eosinophilic asthma patients with flow cytometry. **(B)** Effects of signal pathway inhibitors on the activation of eosinophils in response to PMA/ionomycin stimulation determined by the levels of CD62L, CD63 and CD11b under flow cytometry. *p<0.05, ***p<0.001 and ****p<0.0001. n=7 for **(B)**.

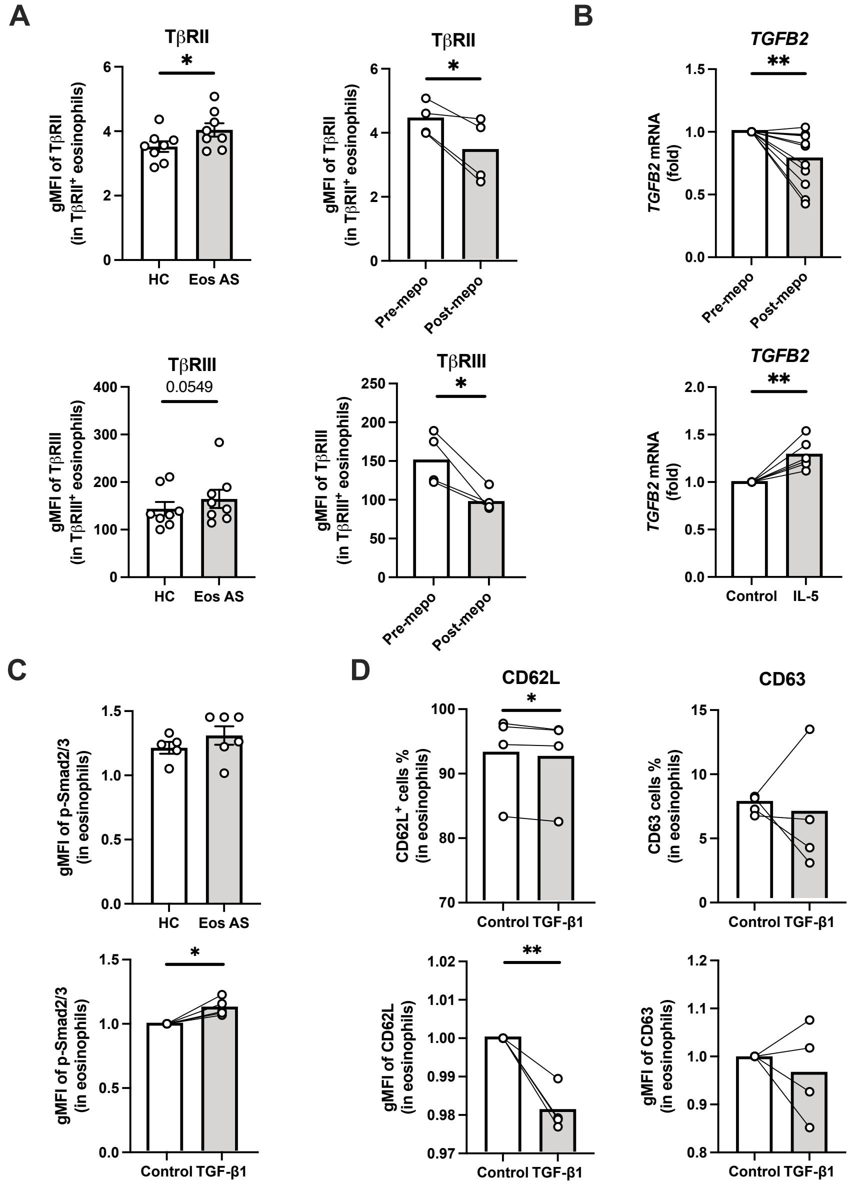

Supplementary Fig. 8 TGF-β pathway in eosinophils from eosinophilic asthma and Its effect on the activation of eosinophils. **(A)** Levels (gMFI) of TGFBR2 and TGFBR3 in TGFBR2⁺ or TGFBR3⁺ eosinophils from HC, Eos AS, Pre-mepo and Post-mepo samples determined by flow cytometry. **(B)** mRNA levels of *TGFB2* in the eosinophils from eosinophilic asthma before or after mepolizumab treatment or from healthy donors before or after IL-5 treatment measured by qPCR. **(C)** Levels (gMFI) of phosphorylation of smad2/3 from healthy control or eosinophilic asthma, or from healthy donors before or after TGF-β1 treatment detected with Phosflow. **(D)** Levels of CD62L and CD63 (ratio of positive cells or gMFI) in eosinophils before or after TGF-β1 treatment measured by flow cytometry. *p<0.05 and **p<0.01.
